# Health data space nodes for privacy-preserving linkage of medical data to support collaborative secondary analyses

**DOI:** 10.3389/fmed.2024.1301660

**Published:** 2024-04-10

**Authors:** Martin Baumgartner, Karl Kreiner, Aaron Lauschensky, Bernhard Jammerbund, Klaus Donsa, Dieter Hayn, Fabian Wiesmüller, Lea Demelius, Robert Modre-Osprian, Sabrina Neururer, Gerald Slamanig, Sarah Prantl, Luca Brunelli, Bernhard Pfeifer, Gerhard Pölzl, Günter Schreier

**Affiliations:** ^1^Center for Health and Bioresources, AIT Austrian Institute of Technology, Vienna, Austria; ^2^Institute of Neural Engineering, Graz University of Technology, Graz, Austria; ^3^Ludwig Boltzmann Institute for Digital Health and Prevention, Salzburg, Austria; ^4^Institute of Interactive Systems and Data Science, Graz University of Technology, Graz, Austria; ^5^Know-Center GmbH, Graz, Austria; ^6^telbiomed Medizintechnik und IT Service GmbH, Graz, Austria; ^7^Department of Clinical Epidemiology, Tyrolean Federal Institute for Integrated Care, Tirol Kliniken GmbH, Innsbruck, Austria; ^8^Division for Digital Health and Telemedicine, UMIT TIROL—Private University for Health Sciences and Technology, Hall in Tyrol, Austria; ^9^Tirol Kliniken GmbH, Innsbruck, Austria; ^10^Department of Internal Medicine III, Cardiology and Angiology, Medical University of Innsbruck, Innsbruck, Austria; ^11^Tyrolean Federal Institute for Integrated Care, Tirol Kliniken GmbH, Innsbruck, Austria

**Keywords:** data-driven healthcare, privacy-preservation, record linkage, advanced analytics, interoperability, machine learning, artificial intelligence, European Health Data Space

## Abstract

**Introduction:**

The potential for secondary use of health data to improve healthcare is currently not fully exploited. Health data is largely kept in isolated data silos and key infrastructure to aggregate these silos into standardized bodies of knowledge is underdeveloped. We describe the development, implementation, and evaluation of a federated infrastructure to facilitate versatile secondary use of health data based on Health Data Space nodes.

**Materials and methods:**

Our proposed nodes are self-contained units that digest data through an extract-transform-load framework that pseudonymizes and links data with privacy-preserving record linkage and harmonizes into a common data model (OMOP CDM). To support collaborative analyses a multi-level feature store is also implemented. A feasibility experiment was conducted to test the infrastructures potential for machine learning operations and deployment of other apps (e.g., visualization). Nodes can be operated in a network at different levels of sharing according to the level of trust within the network.

**Results:**

In a proof-of-concept study, a privacy-preserving registry for heart failure patients has been implemented as a real-world showcase for Health Data Space nodes at the highest trust level, linking multiple data sources including (a) electronical medical records from hospitals, (b) patient data from a telemonitoring system, and (c) data from Austria’s national register of deaths. The registry is deployed at the tirol kliniken, a hospital carrier in the Austrian state of Tyrol, and currently includes 5,004 patients, with over 2.9 million measurements, over 574,000 observations, more than 63,000 clinical free text notes, and in total over 5.2 million data points. Data curation and harmonization processes are executed semi-automatically at each individual node according to data sharing policies to ensure data sovereignty, scalability, and privacy. As a feasibility test, a natural language processing model for classification of clinical notes was deployed and tested.

**Discussion:**

The presented Health Data Space node infrastructure has proven to be practicable in a real-world implementation in a live and productive registry for heart failure. The present work was inspired by the European Health Data Space initiative and its spirit to interconnect health data silos for versatile secondary use of health data.

## 1 Introduction

Real-world data (RWD) is typically gathered over a patient’s lifetime for the purpose of patient care (*primary use*). However, beyond its original use, RWD can be used for other analyses (*secondary use*) to generate additional real-world evidence (1). Among other aspects, secondary use proved to be valuable for cost-effectiveness analysis (2), data exploration (3), clinical outcomes research (4, 5), data validation (6) and data aggregation (7). However, medical data is sensitive by nature. Strict legal frameworks around highly sensitive data impose challenging demands on data holders (e.g., healthcare organizations). On top of that, as opposed to RWD, collecting data in clinical trials is eminently expensive and the resulting data is therefore highly valuable to those who hold it. Both privacy and security considerations as well as the associated costs of health data make data holders exceedingly reluctant to share any data with a health ecosystem. Sharing data also has implications regarding data sovereignty (i.e., who owns and controls data). This is further complicated by the fact, that many countries have not yet fully defined ownership of medical data in their legal frameworks (8). Consequently, health data of different sources is often kept in isolated data silos, and its value for further secondary analyses remains underutilized (9, 10). Connecting silos can accomplish both *vertical linkage* (i.e., more data for one patient) as well as *horizontal linkage* (i.e., more patients for specific data) and thus provide more holistic views on patients and diseases increasing the data’s value for research even further.

An example of secondary use of health data was an analysis of data from *HerzMobil Tyrol* (HMT), which is a telehealth-supported disease management program for heart failure patients in Tyrol, Austria for which patients are recruited after an episode of acute heart failure and receive optimized disease management care by a network of health professionals (11). In HMT, patients are given measurement equipment (e.g., a bodyweight scale, blood pressure cuff), which is connected to an app, through which patients can record daily physiological (e.g., bodyweight), fitness (e.g., steps per day) and self-reported (e.g., wellbeing) data. In Tyrol, over 1,000 patients have been monitored by this telehealth system and the data is highly valuable for secondary analyses. To investigate the clinical effectiveness of the program, electronic medical records (EMR), and clinical outcome data from HMT patients and a control group were compiled for a secondary use analysis (5). For this analysis, data from three different sources were required: (1) telehealth data from the HMT system itself, (2) EMRs from the patients’ hospitals’ information system and (3) information about time and cause of death from Austria’s national register of deaths. This resulted in an aggregated dataset containing more than 80 variables and while reduced mortality for patients in the telehealth program compared to conventional care has been found (5), several challenges were encountered:

- Data linkage—The analysis required linkage of data from three different data sources including hospital information systems (HIS), the *HerzMobil* telehealth system, and the Austrian register of deaths. Data linkage had to be done manually, as the data sources did not share a unique alpha-numeric identifier. Additionally, although the laboratory information systems were part of the same hospitals, they also used their specific identifiers.
- Privacy preservation—To achieve privacy preservation, personally identifiable information (PII) had to be manually removed from the datasets.
- Unstructured data—RWD data used in the analysis contained both structured and unstructured data. The latter imposed additional challenges for the de-identification of text for secondary use.
- Interoperability—While data sources provided coded data (e.g., ICD-10 codes) for various data elements, they did not adhere to one harmonized coding vocabulary or a common data model for the resulting dataset making the individual data sources not interoperable.
- Collaboration—Different data sources and different data types (e.g., unstructured data) required a team of researchers compiling the aggregated datasets using various analysis pipelines, which made intensive communication and exchange of intermediate results necessary.
- Traceability—With more than 80 variables involved in the analysis, tracing all involved algorithms and processing steps used to derive a specific variable proved to be difficult.
- Extensibility—Necessity for both *vertical linkage* of more data sources from out-patient domains as well as *horizontal linkage* of data for comparison with identical *HerzMobil* systems in the states of Styria and Carinthia to improve the data analysis was identified for future studies.
- Automation—To increase repeatability, having the possibility to easily rerun analyses on a regular basis is required. This was not possible with the aforementioned manual labor required.

Comparing the experience from this retrospective view on the challenges encountered during the HMT effectiveness analysis with published literature, a general trend of similar, regularly occurring problems can be observed. Privacy, interoperability, data governance, organizational coordination, data quality and funding considerations are frequently being mentioned as the most pressing issues (12–15). A more detailed view on these challenges is given in the following list.

### 1.1 Privacy, security and data linkage

Health information is highly sensitive data and therefore access is regulated through data protection and security frameworks. To mitigate the data’s sensitivity and to keep with the spirit of the EU’s General Data Protection Regulation’s (GDPR) (16) principle of data minimization, any identifying elements not required for analysis (e.g., names, specific date of birth) should be removed from the dataset in advance. However, removing this information complicates record linkage, which is necessary to associate data with the correct individuals across different contexts and to avoid duplication of subjects. Furthermore, medical free texts (e.g., clinical messages, nursing documentation) typically include references to personal information (e.g., names, addresses) that also infringe on patient privacy and increase the risk of re-identification.

### 1.2 Standardization and interoperability

While interoperability might not be of utmost importance when working with isolated data silos, it becomes a core necessity when connecting data from multiple silos. Source systems store data in different data formats (i.e., data models) and use different vocabularies and thus datasets are frequently not interoperable originally. This requires time-consuming, manual effort to map different elements from the sources into a common dataset (i.e., a feature matrix) and to translate values into a mutual standard vocabulary.

### 1.3 Data quality and availability

As health data is often entered or administered manually, source data needs to be verified to avoid erroneous data. Furthermore, related to the aforementioned interoperability aspects, some elements of data are ambiguously encoded or worded. Also, in some instances, not all source data is available in digital form or complete at all times. These factors require regular contact with data holders for clarification. Lastly, sometimes additional context is necessary for analysis (e.g., labels for supervised machine learning), which is also time-consuming and is known to be associated with it its own unique challenges (17).

### 1.4 Stakeholder management and data sovereignty

Data linkage requires collaboration of multidisciplinary teams of clinicians, nurses, administrators, and engineers. These groups have different interests (e.g., data sovereignty, workload management) that need to be aligned. Dedicating medical and engineering staff to set up, provide and maintain infrastructure to link and harmonize data is generally associated with costs (18). Since health infrastructure projects are often non-profit oriented and executed with public funds, a certain political and institutional will is often required, Also, as data can originate from different sources, datasets can be subject to different data sovereignty spheres and legislation.

### 1.5 Collaboration during data analysis

In complex real-world scenarios, multiple data engineers, data analysts and machine learning engineers are working on the same data. This requires extensive communication and coordination to avoid redundant work on data processing, feature engineering and model development processes. Many experiments require the same standard data and feature engineering algorithms, which are at risk of being duplicated by multiple team members, which ultimately results in less efficient collaborative analysis. To improve collaboration, the concept of feature stores has gained popularity recently (19). The idea is to collect feature extraction algorithms over multiple experiments to nurture a growing repository of re-usable features, which can be made accessible for all team members to speed up machine learning analyses. Additionally, machine learning operations (MLOps) to aid in model deployment requires suitable infrastructure. Kim discussed the software engineering difficulties concerning MLOps, such as complex software stacks and distributed data (20). Due to the intricacies of MLOps for health data, Khattak et al. introduced the term “*Machine Learning Healthcare Operations*” (MLHOps) (21).

Tayefi et al. (14) concluded that key infrastructure technology to facilitate secondary use of health data addressing these challenges is required but still underdeveloped. A typical approach to implementing such infrastructure is the introduction of an enterprise data warehouse or integrated data repository (IDR). Gagalova et al. (22) have described architectural principles of IDRs in the clinical domain distinguishing centralized approaches (General architecture), biobank-driven architectures and federated approaches. They also identified the need for a common data model (CDM) to represent data. Solutions following these approaches are described in literature. For example, *DataSHIELD* is a federated platform by an international consortium of researchers that facilitates distributed analysis to avoid data exchange entirely with a client–server infrastructure for data analysis (23). The *Personal Health Train* (PHT) is another federated infrastructure solution to reuse medical data for secondary use (24). The *PHT* aims to establish FAIR data stations that can be governed by data holders and accessed by analysts whereas trains travel from station to station carrying algorithms that are executed in the FAIR data stations. Secure multiparty computation (25–27) and more recently, blockchain-based concepts (28–32) have also gained popularity to increase data security in privacy-preserving trustless systems. Although keeping data distributed across multiple sources is privacy-minded, performance of machine learning models still suffers in federated learning settings compared to conventional centralized learning (33–35). Therefore, another architectural approach is to accumulate data in a centralized point (i.e., a clinical data warehouse) with secure and privacy-oriented infrastructure. Wirth et al. (36) and Jin et al. (37) both provide a comprehensive overview and analysis of a selection of privacy-minded data sharing networks in their works. CDMs are important for data warehouses to serve as a common denominator when multiple heterogenous data sources are to be linked and standard vocabularies ensure interpretability of data values. A specific successful example of medical data sharing is the open-source software platform *informatics for integrating biology and the bedside* (i2b2) developed by Harvard Medical School (38) to drive clinical research. The partnership between *i2b2* and *tranSMART* (39), an open-source data warehouse developed by a consortium of private pharmacological companies resulted in the *i2b2 tranSMART* foundation (40). Further literature examples include *GIFT-Cloud* (sharing medical image data) (41), the *Shariant* platform (sharing clinical genetic data-testing data) (42) and *IMPROVE-PD* (sharing peritoneal dialysis data) (43). However, it has been outlined clearly that many currently existing solutions are limited to one specific use case (44). Gruendner et al. made use of best-practice principles and established the *KETOS* platform, which is a containerized (Docker) solution with standard vocabularies (SNOMED & LOINC) and the Observational Medical Outcomes Partnership common data model (OMOP CDM) for a more general development environment (44).

While these solutions work well for their intended purposes, they do not completely fulfill our requirements. Blockchain-based distributed systems are proven effective in multiple studies (28–32), however suffer from the slow pace at which this technology is adopted in the health sector, which ultimately makes them impractical currently. While *DataSHIELD* is an excellent example of a framework that enables federated analyses, it is not intended to also support machine learning (e.g., federated learning). The *PHT* is based on data trains containerized with Docker to be sent to data stations where code is executed. In our experience, system administrators of healthcare organizations are hesitant about this form of code execution on their environments even though there are containerized, mostly because they lack control over the code and thus data sovereignty becomes a concern. Furthermore, although the *PHT* could support a form of federated learning, studies have shown, that performance of ML models trained by federated learning can trail behind centrally trained models (33–35). Therefore, for optimal AI applications, data is required to be aggregated in a central point to train models to their full potential, for which key infrastructure is required. While the *KETOS* platform aims to fulfill exactly that, in *KETOS*, privacy and security by limiting data storage to remain within a hospital information system. Therefore, linkage to other data sources is restricted, which is a key requirement for our system.

In this study we propose a federated node-based system architecture called Health Data Space (HDS) nodes. These nodes aim at facilitating linkage (*horizontal* and *vertical*) between multiple, decentralized data sources. The architecture supports privacy-preserving record linkage (PPRL) and additional de-identification algorithms. For interoperability, we outline how we harmonized heterogenous data into the OMOP CDM, which is suitable since our data is mostly observational health data. We further propose how a multi-level feature store can be realized to support collaborative data analytics. We also present preliminary experiments to assess the nodes’ feasibility of supporting MLOps in future developments. We hope to utilize this solution to facilitate time-efficient analyses to answer clinical research questions (e.g., efficiency, health economics) quicker and allow data linkage to scale with related systems (e.g., *HerzMobil Styria* and *HerzMobil Carinthia*).

As a proof-of-concept, we describe a real-world application of a heart failure registry established in Austria with HDS nodes with three different data sources. We further discuss the organizational considerations of developing such multidisciplinary infrastructure. In particular, the following contributions are to be highlighted.

#### 1.5.1 Pseudonymization concept and free text de-identification

To adhere to strict legal frameworks like GDPR, respect patient privacy and minimize risk of exposure, the HDS nodes use a PPRL system to avoid storing quasi-identifiers. In this spirit, an additional de-identification algorithm is in place to remove identifying references from free text data, while aiming to retain context by applying basic entity recognition logic.

#### 1.5.2 Multi-level feature store based on the OMOP CDM

A feature store based on the OMOP CDM is used to avoid repeated feature engineering and improve experiment repeatability. The feature store allows features on multiple levels (e.g., on patient level like age and sex, but also on daily observational level like blood pressure). These features can then be linked into a feature matrix and accessed for later ML experiments.

#### 1.5.3 Case study of sharing secondary data in a heart failure registry

The HDS nodes are used in a real-world case study for a registry for chronic heart failure patients, in which health data from three different sites are linked.

## 2 Materials and methods

**Figure 1:**
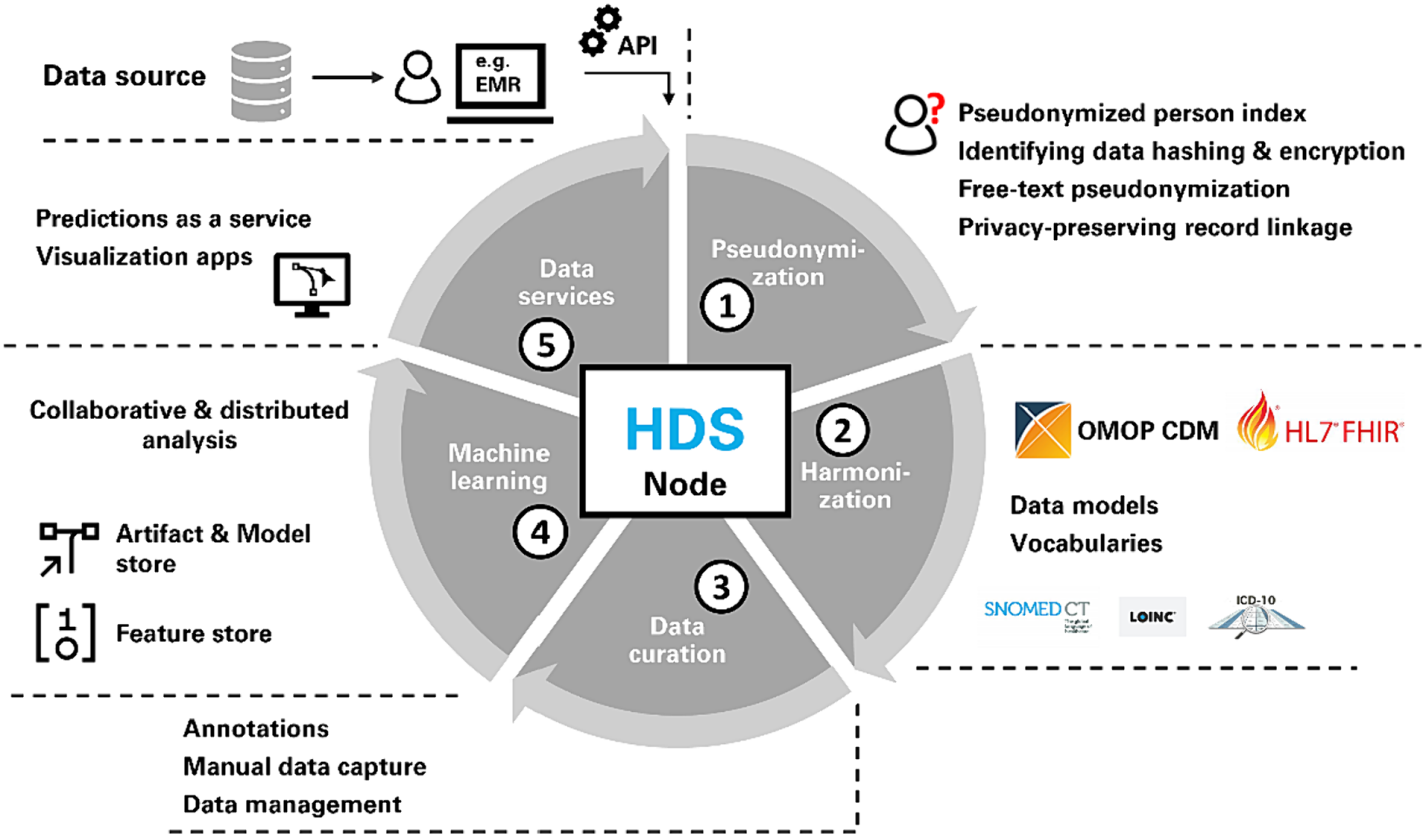
Each health data space (HDS) node consists of the same capabilities for pseudonymization, harmonization, data curation, machine learning support and data services.

We introduce the concept of HDS nodes as fundamental building blocks of health data spaces. The HDS node components are illustrated in Figure 1. Python and the Django Web framework for server components (45) were chosen due to the large popularity of Python in data analysis. During development, only modules and libraries were selected that allowed HDS nodes to be infrastructure-agnostic, meaning they are compatible with deployment on different cloud environments (e.g., Microsoft Azure or Amazon AWS), but can also be deployed on-premises. They also support a variety of relational databases (e.g., MySQL, PostgreSQL). For evaluation, PostgreSQL was used as primary database technology.

Data can be submitted from a source to an HDS node by data holders via a public application programming interface (API) which forwards the data to the HDS node’s Extract-Transform-Load (ETL) framework. The ETL framework consists of a collection of individual ETL classes, that act as converters and first pseudonymize and then transform incoming data into the OMOP CDM. ETL classes are implemented as plain Python classes. No visual editors are used, but instead all steps in the workflow are expressed as code, that digests new data submitted to a node. Data submission can either be automated in regular intervals (e.g., via cron jobs) or manually executed on demand. The data engineering pipeline as seen in Figure 1 starts with pseudonymization (1), which is followed by harmonization (2) after which data is saved in a data store based on the OMOP CDM. We chose the OMOP CDM because (a) it is increasingly adopted in clinical research for observational health data, (b) it provides a large variety of standard terminologies, and (c) it is based on a comparatively flat data model. Data curation services (3) allow for (a) manual data entry through an HL7 FHIR-based electronic data capture system (EDC) and (b) manual annotation and labeling of data. To facilitate ML, a feature store (19) and a model store are implemented for collaborative analysis (4). Finally, data services (5) support the creation of data and visualization apps as well as providing predictions as web services to other applications (e.g., used for primary use of health data). The last part (data services and model deployment) is mainly focus of future work and largely out of scope of this study as further work to mature this aspect is still needed. A data node may use all these components or only a subset of the functionalities. The individual parts of the data engineering pipeline are described in detail in the subsequent chapters.

### 2.1 Pseudonymization

For the pseudonymization component, we expanded the work of the European Patient Identity Services (EUPID) (45), introducing a hash-based pseudonymized person index for patients and healthcare professionals. We further identified clinical sites as additional entities that require pseudonymization. All entity types that are pseudonymized in the HDS nodes are listed in Table 1.

**Table 1:** Entity types of the D4Health Heart Failure Registry that are pseudonymized into master records.

Master entity record	Identity traits
Patients	First name, last name, date of birth, social security number (if available)
Healthcare professionals	First name, last name, date of birth
Clinical sites	Clinical site’s name (e.g., a center, department)

Every record (e.g., patient, clinician) has specific identity traits that uniquely identify them. For pseudonymization, they are transformed into record-level hashes by concatenating the string values of all traits to one large string and applying a hash function to the result. A variety of record-level hash algorithms are already provided by EUPID (including HMAC512, Argon2, Bloom filters) and could be used in the HDS nodes. However, to enable similarity matching, we use locality-sensitive cryptographic long-term key (CLK) Bloom filter (BF) hashes (46). To ensure scaling performance in large networks, we applied MinHash (47) in combination with the Bloom filters. With this blocking strategy, hashes are only compared to the most similar ones instead of all available hashes. This drastically reduces the amount of redundant Bloom filter comparisons, which can get computationally expensive once large quantities of records are available. Identity traits are hashed into a 459-bit BF vector and then associated with a randomly generated alpha-numeric pseudonym. As an additional layer of security, HDS nodes operate two independent databases: One to store the actual health data from the data sources without personal data (i.e., the data store) and a separate one to store pseudonymized identity traits (i.e., the pseudonymized person index). The link between data and identity traits is achieved via the alpha-numeric pseudonym, which is available in both databases. As an additional layer of privacy, all records (e.g., patients) are given context-specific pseudonyms (i.e., one pseudonym per node). For example, a patient will have pseudonym *P1* in one node, pseudonym *P2* in another and if both data sources for this patient are linked in a central node, will be assigned pseudonym *P3*. While this connection is traceable in the person index, it will not be visible for data scientists only working with the health-related data. To increase security, BFs are encrypted at rest in the database using AES256 encryption. The encryption key and the HMAC keys required for BF generation are stored outside the databases. For record linkage, the Jaccard distance is applied to all possible pairs of BFs in the person index to identify potential duplicates. Depending on a threshold decision, full matches and partial matches (e.g., typographic errors) are identified and logged. While full matches are automatically consolidated, partial matches are flagged to be resolved at the data source by human administrators to ensure correctness.

Pseudonymization is also applied on free text data (e.g., clinical notes) with an advancement of a previously developed algorithm (48), which relies on name dictionaries (public and internal), common precursors for names and regular expressions to remove personal references such as names, phone numbers, locations, addresses, email addresses and websites. Public name dictionaries were scraped from Wikipedia articles of category *Person* and the publicly available search tool for physicians in Tyrol. The internal dictionary is comprised of all names within the available data sources. A basic rule-based entity recognition is applied to retain context after removing potentially valuable information by de-identification. The entities of *healthcare professional*, *patient*, *person*, *location*, *phone number*, *e-mail address*, *address*, *ZIP code* and *website* are recognized, and corresponding pseudonyms are assigned, which are consistent throughout the entire text corpus.

### 2.2 Harmonization

For each data type or dataset that is to be digested into the data store, individual harmonizing ETL classes must be developed manually in advance. In essence, these harmonizer classes read the data they are designed for and map data points to suitable OMOP CDM fields. For further interoperability, the ETL classes also map values of data to standardized vocabularies of the ICD-10, SNOMED-CT, LOINC and ATC terminologies. Any data that is processed like this by an ETL class is tracked to enable version control for the data store. These ETL classes can either be integrated into the HDS node to populate data automatically into the data store if the corresponding data is regularly updated, or resort to outside ETL processes if data is simply imported once without expected regular updates. Mapping all incoming data into the OMOP CDM with standard vocabularies created a scalable data store that can be extended should any new data sources be connected to the HDS node.

### 2.3 Data curation

The ETL process framework is mainly intended for importing and harmonizing of RWD from primary data sources (i.e., the data’s origin). On many occasions, additional data is collected that does not originate from the primary care system, such as quality-of-life data [e.g., MacNew questionnaires (49)]. For this reason, we implemented a basic electronic data capture (EDC) system. As each HDS node provides a FHIR repository, we used FHIR Questionnaires to define EDC forms and FHIR CarePlans to express typical workflows. Entered forms and their completion statuses are stored as FHIR Questionnaire responses. The EDC component is tied to the pseudonymization component, so that patients can be registered manually and linked to existing patients from primary care data sources with the record linkage algorithm. For enhanced privacy, subjects in the EDC system receive their own pseudonym which is automatically linked to the pseudonym used in the OMOP database. Both the FHIR Questionnaires as well as the FHIR Questionnaire responses are transformed via ETL classes into the OMOP CDM. We defined functions that transform them into the OMOP entities *VisitOccurrence* (the action of completing a form), *SurveyConduct* (details on the questionnaire itself) and *Observation* (the actual questions) and store them in the OMOP database.

Our experience with HMT has shown that some critically valuable data is only available in unstructured form. For instance, in the telemonitoring setting of HMT, physicians and nurses make extensive use of free text notes to capture additional insights into patients’ condition and treatment. Similarly, in the patients’ EMR, discharge letters contain free text diagnoses and discharge medication prescriptions. Based on previous work (50), we integrated (a) a tool to create annotation corpora from OMOP data, and (b) a multi-annotator tool for manually annotating text data on both the sentence and the full-text levels. Annotated corpora can be accessed through APIs like data from the data store for further analyses (e.g., training classification algorithms).

### 2.4 Collaborative analyses

**Figure 2:**
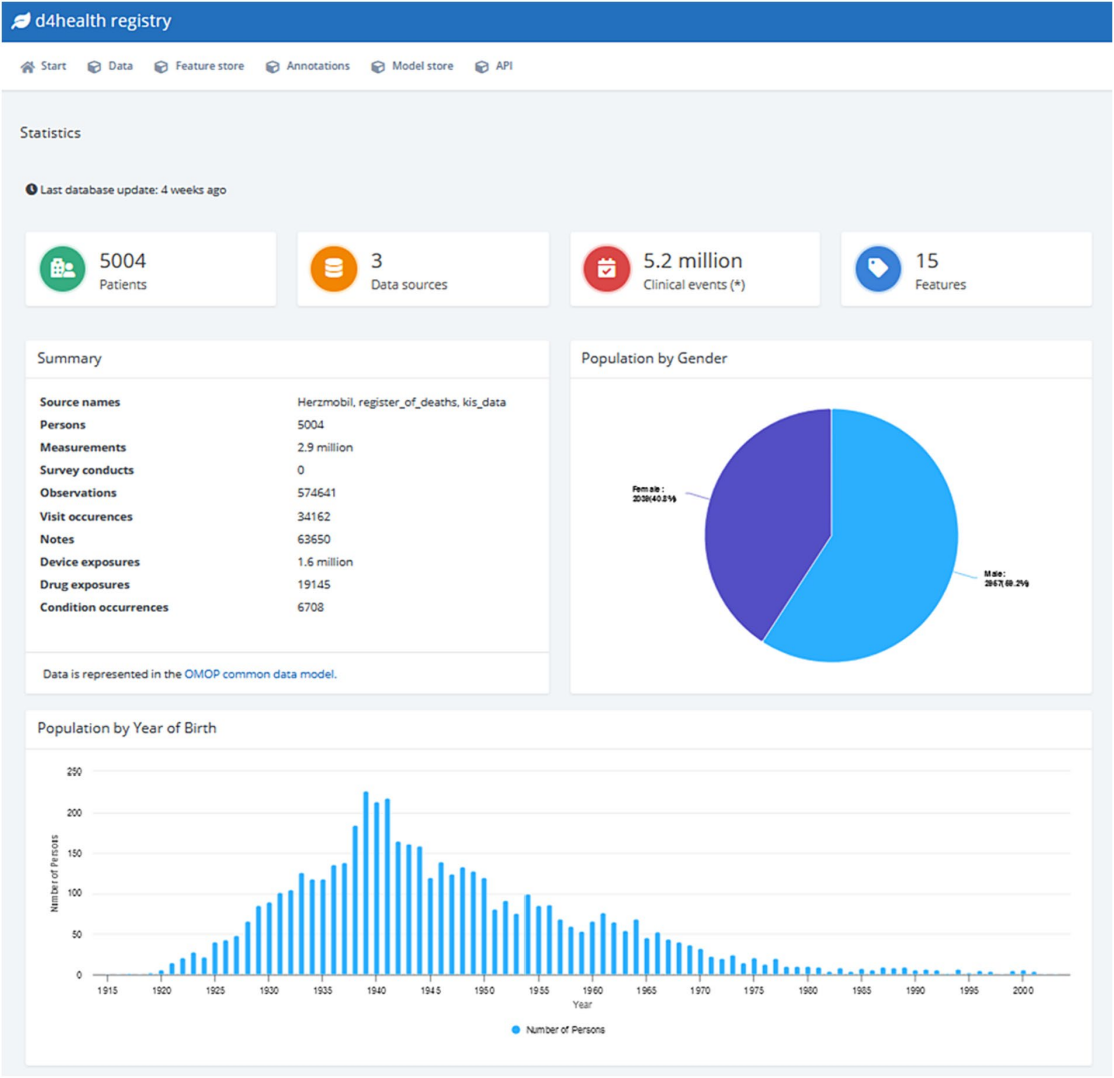
Screenshot of the default dashboard of the D4Health Heart Failure Registry.

**Figure 3:**
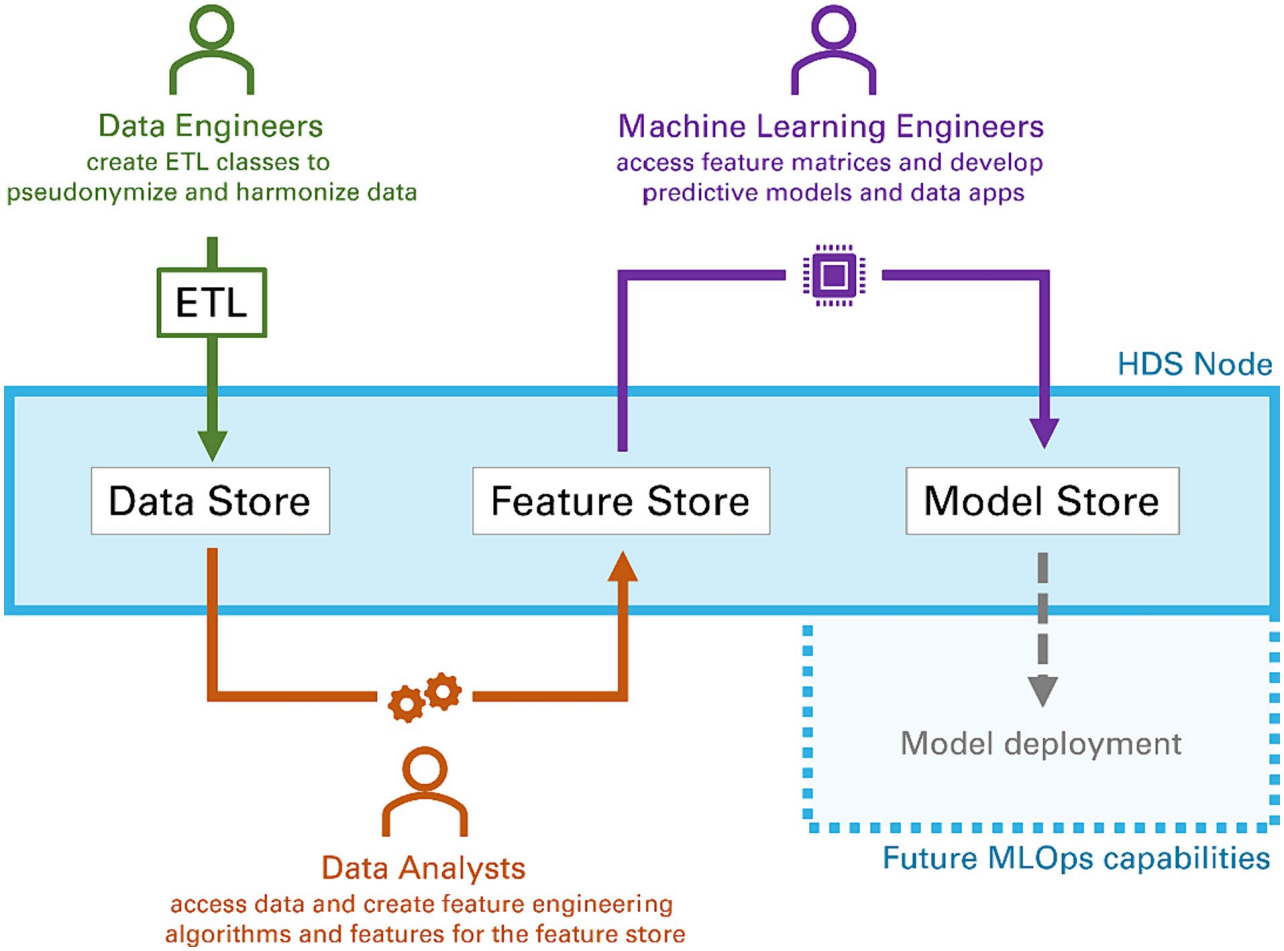
Typical workflow of different data scientists (data engineers, data analysts and machine learning engineers) collaborating within the components of a Health Data Space node to process raw data, extract features and develop models. The final step model deployment is subject to future work.

Typically, data analysis and ML tasks are complex, iterative processes with multiple steps involving an interdisciplinary group of experts (20). Depending on their specific role, experience, and training, team members might prefer different tools (e.g., Python, MATLAB). To support the usage of said tools, the HDS nodes provide a dedicated API to extract pseudonymized data via SQL queries from the nodes’ data store. We developed functions for Python, R and MATLAB to (a) access an HDS node’s data store via the API, and (b) transform the received data into native data formats, including *Pandas DataFrames* (Python), *data.frames* (R), and *tables* (MATLAB). This platform-agnostic way of accessing data allows data scientists to rely on their preferred tool chain they are familiar with to develop algorithms and models. When given access to an HDS node through the permission management system, data scientists can browse the data available (see Figure 2 for an example) and simple descriptive statistics (e.g., distribution of sex and age) are provided via a dashboard. An SQL editor allows data scientists to understand the database scheme and test SQL queries before executing them in their processes. SQL queries are tracked for audits and can be saved for repeated executions. Data scientists are also given access to a collection of already developed feature extraction algorithms, called feature store. Figure 3 illustrates how a typical workflow involving feature generation, model development and model deployment involving a data engineer and a data scientist could be executed.

#### 2.4.1 Feature store

Once the required data is extracted via API queries, data analysis often requires the calculation or engineering of features (i.e., derived values from raw data). These represent information-dense data points to be used for machine learning modeling. Since medical datasets are relatively sparse, typically multiple people work on the same data. However, on occasion, different analyses by different data scientists can require the same features. For example, with the available blood pressure data of systolic and diastolic values, it will often be required to calculate the pulse pressure. The nodes’ feature store allows data scientists to upload the algorithms’ code they have developed into a so-called feature store. The feature store’s main purposes are first, to reduce the risk of repeated developed of the same feature engineering algorithms and second, to provide future analysts with a large number of useful features already developed by other team members, that grows over time. This should facilitate collaborative and efficient data analysis. At the time of writing, the feature store supports feature development in Python. Feature engineering algorithms are documented (e.g., author, date, description), versioned and deployed within HDS nodes. Any feature generators uploaded into a node are quarantined initially and only deployed after an audit by an administrator for any malicious code.

Features can be calculated on different levels (e.g., daily level like blood pressure, patient level like height). The OMOP CDM already supports features related to the patient-level including source code for feature generation based on the *CohortDefinition* entity and its associated attributes (*AttributeDefintion*). For the feature store, we extended this functionality to support features on other levels and to support further meta data (e.g., author, timestamp, source code, technology, description). Each entity in the OMOP data model (e.g., *Person*, *Observation* or *Measurement*) has a counterpart in the feature store so that features can be calculated on *Person-level* (e.g., number of re-hospitalizations in the last 3 years), on *Observation-level* (e.g., daily medication adherence) or on *Measurement-level* (e.g., blood pressure). The feature store communicates with the data store, and is notified of all data updates, so that features are re-calculated whenever new data arrives, or existing data is updated. Features are stored in a compact JSON data structure to accommodate use cases with high numbers of variables.

Data scientists can explore available features through a web-based interface. The interface gives a superficial description and overview of each feature (e.g., availability and distribution of values) to give analysts quick insight whether a feature might be useful for their analyses. Features can be accessed through a dedicated API similar to that of the data store. When features of a given level (e.g., *Person-level*) are accessed, all features on this level are aggregated into one feature matrix.

#### 2.4.2 Model store

Analogous to the feature store, HDS nodes also support a model store, which is a collection of models developed outside of the HDS node’s infrastructure. At the time of writing, the model store can digest any model developed with Python’s scikit-learn (51) module via manual upload to the model store by the use of model serialization through the built-in pickle module. These model and their required artifacts are accessible via API. In future, this model store should serve as the basic framework for supporting MLOPs. Models and other data apps (e.g., visualization apps, dashboards) are planned to be deployed in this store to provide specific functionalities as services (e.g., prediction as a service).

### 2.5 HDS nodes in a network

HDS nodes are self-contained units that are linked to one data source (e.g., an EMR or a subsystem) and are pseudonymizing, harmonizing and providing data in an analysis-friendly way. Aggregating health data in one place, thus populating a node with data from multiple sources is particularly difficult if data sources are in different institutions or even countries. We have therefore designed the nodes in a way that collaborators can share artifacts and data according to defined data policies and trust in the system, thus forming a health data space enabling versatile data governance schemes. Healthcare organizations are thus enabled to meet the requirements of local data sovereignty legislation by controlling exactly what data is shared with whom. We have defined 4 layers of sharing elements depending on the level of trust between the nodes (see Table 2). Sharing of elements is done through a dedicated REST API with the HDS nodes’ ETL process framework. For instance, on level 4, an HDS node might share specific raw patient data points with another HDS node. In this case, the corresponding ETL process can be activated to allow sharing as long as valid endpoint and credentials for the other HDS node are provided. While the ETL process itself is still executed locally at the source’s node (transformation into an OMOP observation), its results are relayed to the other HDS node where they are stored. For levels 2, 3 and 4, it is essential that patients existing in both HDS nodes are correctly associated and linked. Therefore, both HDS nodes must agree on (a) a common set of identity traits and (b) a certain hashing strategy, including related secrets (e.g., a secret key in case of Bloom filters).

**Table 2:** Information sharing options depending on level of trust.

Trust level	Requirements	Sharing	Possible use cases
1: Artifacts	-	A data node can share artifacts (e.g., feature extraction algorithms or trained models) with other data nodes.	Sharing algorithms for a federated analysis task. Data itself stays in the HDS.
2: Feature information	PPRL strategy needs to be aligned	A data node may share information (e.g., which features are available) and extraction algorithms of generated features	Increasing findability of data of interest for participating network partners, which then can specifically requested or consent can be requested.
2: Features only	PPRL strategy needs to be aligned	A data node may share generated features with other data nodes	Aggregating selected data from the same patient population in a single place without revealing the raw data (e.g., a node might extract data from clinical notes and only provide extracted data without revealing the clinical notes themselves)
4: All data	PPRL strategy needs to be aligned	OMOP CDM data can be shared	Aggregating data from the same patient population in a single place.

### 2.6 Evaluation in a real-world application

The HDS nodes and various configurations can be helpful in different use cases. We explored the feasibility of the HDS node solution in a real-world scenario in the Austrian federal state of Tyrol, connecting data sourced from three origins (one healthcare organization, one telehealth system and Austria’s national register of deaths) into a registry for heart failure patients. To evaluate the architecture’s readiness to deploy ML models in the future, a simple use case of a natural language processing (NLP) experiment was tested. For this, free text messages exchanged between healthcare professionals and patients from HMT were de-identified. This de-identification was based on an improved algorithm of a previously developed pseudonymization algorithm (48), which removes meta data (i.e., author, corresponding patient) and identifying references from the corpus (e.g., names, addresses) from the texts. This algorithm was evaluated on a stratified subsample of 200 messages. Subsequently, messages were annotated by human experts and an ML classification model based on Latent Dirichlet Allocation (52) was trained. The model was deployed on the network and the result was presented via a web service based on the open-source visualization library Dash (53).

## 3 Results

Three main results are presented in the following chapters: (1) the four levels at which data can be shared depending on the level of trust of the participating partners in an HDS node network, (2) a real-world case study of an implemented network at the highest trust level and (3) preliminary results from a MLOps feasibility study with an NLP use case.

### 3.1 Levels of trust in an HDS node network

To comply with different expectations and agreements of trust between participating partners, we designed HDS nodes in a way that they enable four levels of possible data sharing (summarized in Table 2):

1. Trust level 4: All data and artifacts (e.g., feature engineering algorithms, models) of a node is shared with all other nodes, including raw data from the data store and all available features. In this setting, data is typically aggregated in a central HDS data node. This use case would be helpful for scenarios, where data from the same patient population is to be aggregated in a single place for centralized machine learning.
2. Trust level 3: While trust level 4 is feasible in a setup where all nodes belong to the same data holder, in a cross-institutional network data holders might hesitate to share their transformed OMOP database. As a result, nodes can form a trust level 3 network. At this level, each node performs pseudonymization on the pre-defined elements of the patient record but keeps the data in the OMOP database locally. In contrast to a trust level 4 network, each node computes its own feature matrix (e.g., on patient-level, on daily-level) and then only shares the results along with the code used to compute the features with the heart failure registry node. This prevents sharing of any raw data from the data store. For example, any features generated from clinical messages can be exchanged for analysis without actually sharing the texts themselves.
3. Trust level 2: If the calculated features from the feature store should also not be shared, a trust level 2 network can be used. At this level, the connected HDS node only provides other nodes with the information, which features it has for a given patient, similar to the FAIR principles. To achieve this, trust level 2 connected nodes participate in PPRL, meaning consistent patient identifiers exist throughout the network. A trust level 2 network can be used to make data more findable for participating partners of the network. If specific data is found, which is required for analysis, partners can contact the corresponding data holders and patients to inquire about consent to access the data.
4. Trust level 1: At the lowest level of trust, no data is exchanged. The connected nodes only inform others that it exists and provides meta data about the contents (i.e., what kind of data is available). For this, also no PPRL across nodes is required. The only shared contents are any produced artifacts. A trust level 1 network could be used as infrastructure for federated analyses by sharing feature engineering algorithms.

### 3.2 HDS nodes in a data-sharing network for a heart failure registry (trust level 4)

The HDS node solution was evaluated in close partnership with tirol kliniken (Tirol Kliniken GmbH). Data from three different sites was extracted to an HDS node, respectively (see Table 3): EMR data from the tirol kliniken’s hospital information system, HMT telehealth data, and an export of Austria’s national register of deaths. Data transfer specifications were defined with cardiologists to select which EMR data elements are required.

**Table 3:** Data sources connected within the D4Health Heart Failure Registry.

Data site	Description	Type of data	No. of ETL processes
tirol kliniken hospital information system	Electronic medical record (EMR) data	Demographic data (age, gender), height, date of admission, discharge and possible readmission, laboratory values from the laboratory information system, diagnoses (ICD-10 coded), NYHA class	8
HerzMobil telehealth data	Daily physiological values measured by patients themselves using medical devices, transmitted to smartphone via Bluetooth and symptoms	Blood pressure, heart rate, bodyweight, medication information (prescription and self-reported intake adherence) and self-reported wellbeing score (“good,” “medium,” and “bad”), clinical notes by physicians and nurses	18
National Austrian Register of Deaths	Export of register of deaths records	Date of death	1

**Figure 4:**
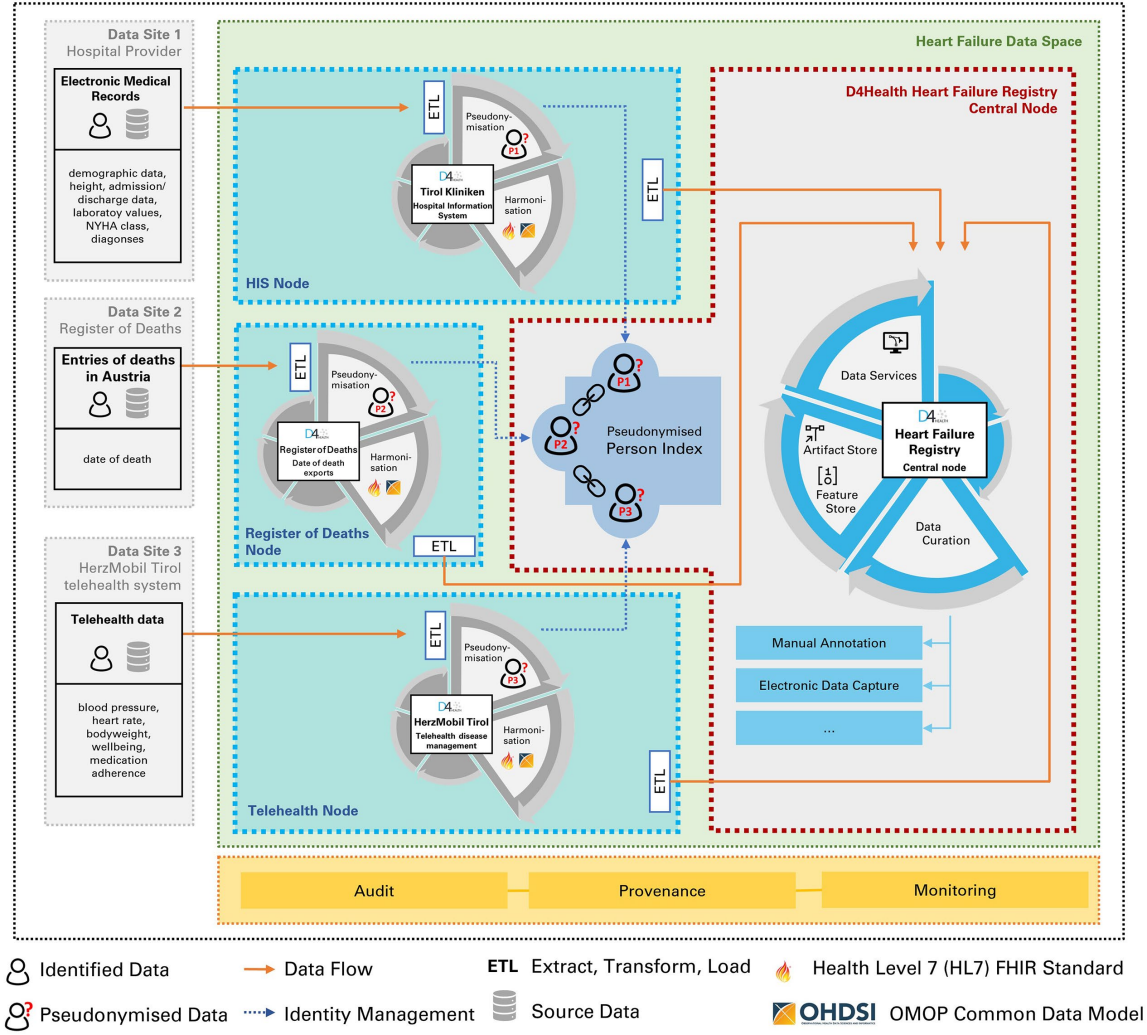
Three Health Data Space nodes (tirol kliniken, HerzMobil Tirol, Register of Deaths) are linked to a fourth, central node, in which the registry is located. Identity management and record linkage is done via the pseudonymized person index.

For these three sites individual HDS nodes were installed, which were linked to a “D4Health Heart Failure Registry,” represented by a fourth HDS node, forming a trust level 4 network (see Figure 4). While the three HDS nodes related to the sources could contain unstructured, identifying data (e.g., discharge letters), only selected, de-identified data was shared with the D4Health Heart Failure Registry HDS node according to the data transfer specifications. In this specific application, the central data node was deployed within the institutional borders of tirol kliniken.

Each node performs pseudonymization of its own identifiers (first name, last name and date of birth of patient, optional social security number where available) by computing a Bloom filter of the corresponding identifier and sharing it with the central D4Health Heart failure registry node. Here, feature ETL classes have been deployed to calculate features.

The HDS node network constituting a trust level 4 network was deployed, operating in a routine care environment and at the time of writing, is continuously linking the data from the three data sources to the registry in a privacy-preserving manner. Record linkage also consolidated duplicated patients. The PPRL found in the *HerzMobil* telehealth node 9 full matches and 19 partial matches, resulting in a duplication rate of 0.70%. The partial matches were subsequently assessed by human experts and found to be all false positives. The hospital information system as well as the Austrian register of deaths nodes had no duplicates since they already used a unique identifier in their respective systems.

At the time of writing, the D4Health Heart Failure Registry HDS node contains data from 5,004 patients, over 2.9 million measurements, over 570,000 observations and more than 63,000 clinical free text notes. In total, over 5.2 million clinical events (i.e., individual data points) are accessible. Figure 2 shows a screenshot of the default dashboard of the D4Health Heart Failure Registry HDS node, which displays basic descriptive statistics to provide an overview of the included data, which can be adapted, according to specific use cases and preferences. To assess performance and scalability, the execution time of individual ETL converters has been recorded. The ETL classes that have transformed most frequent data types were *measurement* (1,173 data points/s), *observation* (1,269 data points/s), *device exposure* (1,445 data points/s), *observation period* (230 data points/s) and *note* (1,151 data points/s). A full list of performance of ETL classes with at least 1,000 data points is presented in Table 4. With increasing amounts of patients, registration slows down significantly as the PPRL framework requires increasingly more comparisons since new patients have to be compared to all registered patients. In our experiments, the application of MinHash (47), increased the speed of registration from 2 per second to 40 per second.

**Table 4:** Performance of individual ETL converter classes with at least 1,000 data points transformed.

ETL class	Data points per second
Visitation	2092.24
Device exposure	1444.89
Drug exposure	1276.43
Observation	1268.78
Measurement	1172.98
Note	1151.05
Condition occurrence	420.14
Observation period	229.87
Person	57.96

As some of the patients also had coronary heart disease (CHD), another node was established to collect quality of life information from them via FHIR Questionnaires. 60 CHD patients were included in a preliminary node, which is not connected to the registry at the time of writing. Patients completed the MacNew quality of life questionnaire at the start of the telemonitoring phase and once again at the end of the phase to track improvements in the quality of life during the program. These FHIR Questionnaire responses are mapped into the OMOP CDM and are planned to be linked into the D4Health Heart Failure Registry in the future.

### 3.3 Feasibility experiment of deployment of a natural language processing model

**Figure 5:**
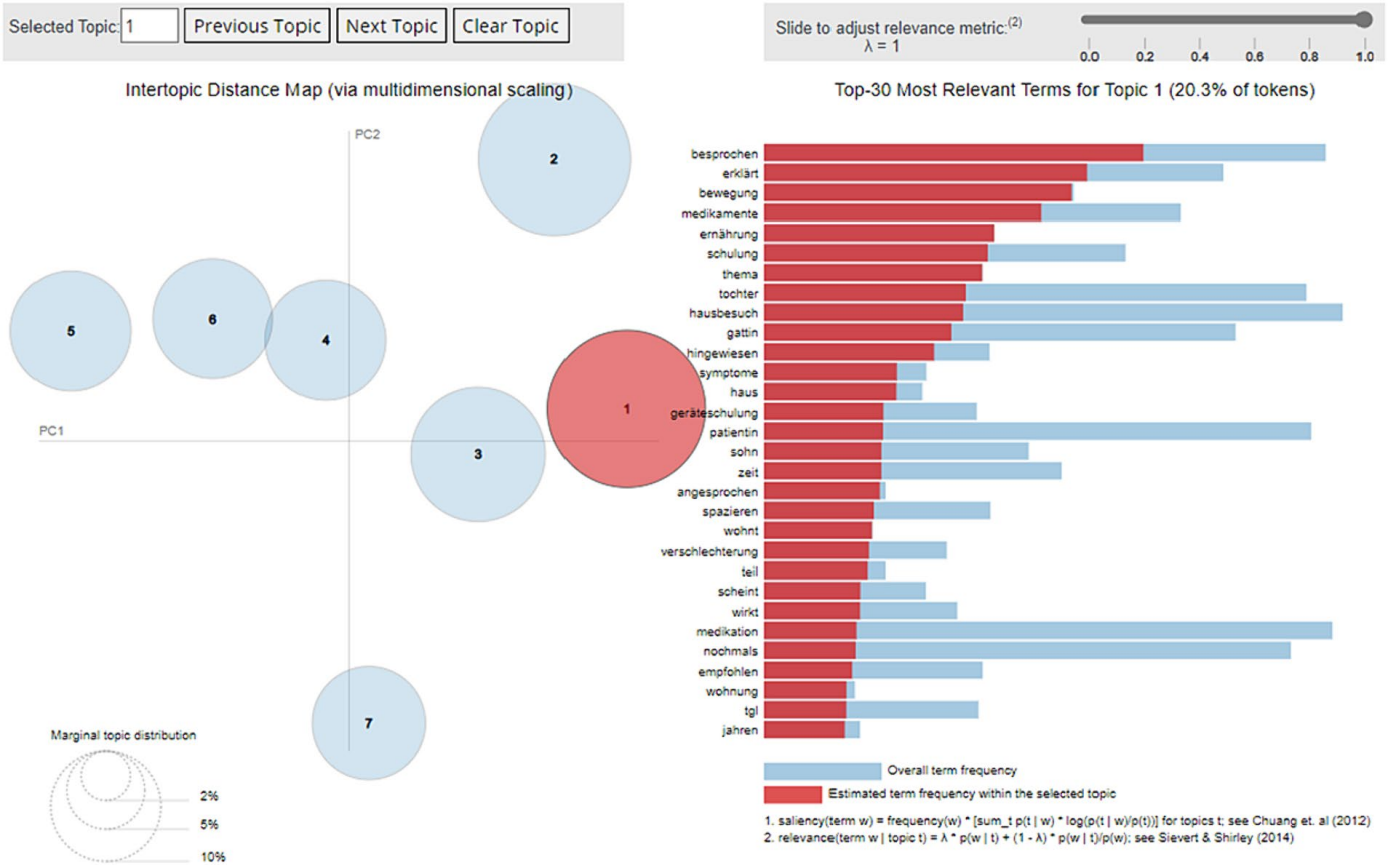
Screenshot of the results of the Latent Dirichlet Allocation model developed from all free-text clinical notes of the telehealth service and deployed to the infrastructure’s central. Circles on the left illustrate the 7 identified topics in an inter-topic distance map via multidimensional scaling. Bars on the right show the top-30 most relevant terms (in German) for the selected topic 1 “council/training” and their frequency within the related topic (red) and overall, within the corpus (blue).

To evaluate the capability of our approach to deploy ML models, a basic NLP use case was successfully executed. The pseudonymization algorithm achieved high performance (accuracy: 93.99%, sensitivity: 0.94, specificity: 0.93). Subsequently, the messages were labeled by 9 expert observers using the HDS node’s annotation tool (50). Finally, the labeled data was extracted via API to a Python development environment, in which the LDA model was trained. The artifacts produced by the model were successfully deployed within the infrastructure and were reachable via API queries from outside with corresponding permissions. A specific visualization tool could successfully be deployed for exploring and quality-controlling the model (see Figure 5).

## 4 Discussion

We presented the Health Data Space nodes as flexible system architecture units, which we evaluated in a real-world application called the D4Health Heart Failure Registry. The results obtained from this case study confirm the infrastructure’s utility. The processes of linking, harmonizing and analyzing data have proven to be functional. Feature engineering and modeling have been explored experimentally and have shown promising, preliminary results in a proof-of-concept natural language processing use case. Extending the functionality of MLHOps (especially model deployment) to industry-level readiness is subject of future research and development.

Although other approaches that address parts of our requirements exist, we hope to contribute new approaches to the complex challenges of sharing and linking medical data with a strong focus on privacy-preservation. *DataSHIELD* enables federated analyses but is not intended to aggregate data into a common feature matrix for centralized machine learning. The *PHT* does that but concerns of containerized code execution with Docker containers make it ultimately nonviable for our application. While other privacy-preserving frameworks were applied to medical data (e.g., *KETOS* platform) and have used Bloom filters [e.g., (54)], linkage assisted with Bloom filters across multiple sources of medical data has not been demonstrated yet. As an additional layer of privacy, we proposed node-specific pseudonyms to avoid using the same pseudonym in multiple contexts, which risk exposing patients by linkage attacks. Privacy-preservation was further focused on by including automated free text de-identification as part of the framework. This is noteworthy, as the rise of large language models (e.g., ChatGPT) has renewed interest in medical free text recently. The application of MinHash (47) with the Bloom filters ensured scalability of the PPRL strategy. To assist in organizational coordination of privacy-oriented data sharing, we introduced four levels of trust within a data sharing network (see Table 2) to provide guidelines for real-world applications. Another novel contribution of our presented architecture is the implementation of a multi-level feature store with the increasingly popular OMOP CDM, which also has not been described in literature. Although, the OMOP CDM supported features on a patient-level with the tables *AttributeDefintion* and *CohortDefinition*, we extended this capability to also represent features that change on a daily basis (e.g., blood pressure).

To summarize our efforts, we combined established techniques (e.g., PPRL, ETL frameworks) with novel ideas (e.g., multi-level OMOP feature store, trust levels, context-specific pseudonyms) to create a starting point for the development of a “full suite” for collaborative analyses of medical data that assists in the entire data science process from start to finish. The HDS nodes have tools for data collection (e.g., FHIR Questionnaires), data cleaning (e.g., de-identification, data annotation), data exploration (e.g., dashboards) and feature engineering (e.g., feature store) and we are aiming to complete the process by implementing sufficient model deployment (e.g., model store) in the future.

To demonstrate the real-world feasibility of this architecture, an ensemble of HDS nodes was applied in a data sharing network for a real-world heart failure registry. Establishing such an infrastructure requires close collaboration between multiple partners, whose interests must be balanced. This concerns not only data governance considerations, but also varying requirements of (a) functionality, (b) processing tools and (c) jurisdiction.

- To address different functionality requirements, HDS nodes are designed in a modular, flexible and scalable way. This not only refers to including data sources currently, but also to apps and services like predictive models and visualization in the future. Linkage to other, similar infrastructures and data sharing with other HDS nodes is supported to different degrees depending on the level of cooperation and trust within a network.
- To enable data scientists to work within their own familiar environments, development of analyses tools is decoupled from the infrastructure. Relying on overly generalized tools can be problematic and enabling data scientists to work with their domain-specific tools is preferable.
- Individual identity holders are able to fully create and control their credentials. Each jurisdiction operating an HDS node is able control the inputs, processing steps and outputs of the node. Data sovereignty is also part the EU’s European Strategy for data (55).

Apart from organizational challenges to coordinate stakeholder interests, we also addressed interoperability on four levels: (1) Syntactic interoperability: ETL processes automatically import and transform source data into the registry. Export functions for JSON, CSV and Microsoft Excel are provided for external use. (2) Semantic interoperability: Data is harmonized using the OMOP CDM. Standard vocabularies are used for further interoperability (SNOMED, LOINC, ICD-10, ATC). (3) Pragmatic interoperability: Linking data also means linking institutions, partners, and pre-existing networks. Data sharing was realized with specifically designed data sharing policies for transparent collaboration process, over which the source data’s managers still have control. (4) Legal interoperability: To comply with legal frameworks like GDPR and ethical considerations, the architecture is based on a pseudonymization and privacy-preserving record linkage infrastructure. HDS nodes can be connected on different trust levels (see Table 2).

Mapping different data structures and models into the OMOP CDM and encoding into the SNOMED vocabulary proved to be a major challenge. For example, the telehealth system included information about prescribed medication usually in brand names as available in Austria. However, SNOMED as an international vocabulary did not necessarily provide these exact names and thus a correct mapping was not always possible. As a workaround, medication was encoded according to their active ingredients (i.e., the chemical compounds). Furthermore, for physiological values from the telehealth system (e.g., blood pressure) multiple SNOMED concepts were available. For example, SNOMED provides multiple blood pressure concepts depending on the body position during measurement (e.g., lying, sitting, standing). However, in the telehealth setting, patients measure data without supervision and thus this information is not available. As a compromise, generic concepts were selected at the cost of minor imprecision. Also, telehealth visitations (e.g., by nurses) were simply not available in the OMOP CDM and thus were difficult to represented within this specific CDM.

### Limitations

The infrastructure is subject to limitations that need to be discussed. Firstly, at the time of writing, the infrastructure’s focus is on observational health data. Other data modalities like time-series, images or genomic data are currently out of scope. Meta data about the D4Health Heart Failure Registry are not made publicly available so far, e.g., via a FAIR Data Point (FDP) as suggested by the FAIR principles (56). Provision of the metadata in an FDP would further improve the visibility and re-usability of the data in the future and enable collaboration with other frameworks (e.g., PHT).

In the presented case study, only one of the sites was a healthcare organization, limiting the scope of the currently demonstrated capabilities. Further, both the EMR data, which is directly from tirol kliniken’s HIS, and the data from the HMT telehealth system, which is operated by a subsidiary of tirol kliniken (the *Tyrolean Federal Institute for Integrated Care*) are domain of tirol kliniken. The central node was operated in tirol kliniken’s institutional infrastructure to avoid raising concerns over data sovereignty. Linking multiple healthcare organizations complicates the task considerably and increases the necessary technical, organizational and legal effort since data is leaving institutional borders. While the presented HDS nodes are designed to also realize such complex settings from a technical point of view, a real-world implementation remains to be demonstrated and is subject of future studies.

To address the issue of data governance and sovereignty, we have segmented access into four levels according to the trust between sharing partners. As requests by data holders can be extremely specific and legislative framework highly intricate, this simplification might not be appropriate for all use cases. A more granular permission and sharing framework would be required to address this fully.

Further, although access to the HDS nodes is possible via APIs from various data science tools, such as Python, R or MATLAB, feature and model deployment is currently only supported for Python. In specific settings, we have already explored model deployment via the Predictive Modeling Markup Language (PMML) between Python and MATLAB, however, this is not yet deployed in the productive HDS node infrastructure. Furthermore, at the time of writing, the model store only supports models developed with scikit-learn (51).

Lastly, although the free-text de-identification performed satisfactorily well (see chapter 3.3) for clinical messages to protect privacy, it is fine-tuned for this application with specific name dictionaries and regular expressions following local rules (e.g., Austrian phone numbers, Austrian postal codes) and therefore will not translate well into other applications.

### 4.1 Outlook

The NLP proof-of-concept use case served as first steps of implementing satisfactory MLHOps support in the HDS nodes. Implementing support for additional commonly used ML and industry-leading frameworks (e.g., TensorFlow/Keras, PyTorch) is subject of future development. Once reliable functions for model deployed are implemented, various other use cases present themselves. Two major groups of data services could be useful, which could be developed outside an HDS node (e.g., a local computer) and uploaded to a node:

1. Model interfaces to provide predictions as a service to healthcare professionals and data scientists. Examples include predicting of major cardiac events, risk stratification of the patient population and outcome prognoses. Another interesting, yet highly specific use case for HMT, would be predicting, which patient would benefit from extending the standard 3 months telehealth disease management program to allocate resources more efficiently. However, further research is necessary to explore the potential of data-driven applications used in the treatment of heart failure patients.
2. Interactive data exploration apps like visualizing dashboards. We provided a basic example with the LDA model implemented with the open-source library Dash (53). Other examples include visual representation of medication adherence or measurement deviations.

Additionally, updates of the HDS nodes based on recent health data can currently either be triggered manually or based on routines in regular intervals. Therefore, any predictions for individual patients would currently face a certain time delay, until all data needed is present in the respective HDS node. Functionalities that trigger data transfers upon updates in the source’s database could be explored further in future development, which would enable real-time predictions.

In the future, we will be investigating the expansion of HDS nodes to support privacy-preserving AI with multiple nodes, focusing on federated analysis, secure multiparty computation, exchange of synthetic data and other promising approaches in addition to PPRL. Federated learning is very appealing in medicine and HDS nodes are especially well-suited for it since they provide uniform distributable nodes with standardized data. Developing models locally, without even centralizing data, has the potential to further increase privacy, security, and trust in the system. An additional advantage might be that it serves as incentive for potential partners to join the network and gain access to well-performing models. Furthermore, partners that only contribute small amounts of data could benefit from the knowledge extractable from larger datasets.

We identify considerable potential for the D4Health Heart Failure Registry specifically in adding additional data sources. Further we aim to test the HDS nodes in an actual cross-institutional data sharing setting in future research. This includes first and foremost other *HerzMobil* systems (e.g., in Styria and Carinthia) for horizontal linkage. Furthermore, vertical linkage by including cardiac implantable electronic devices is especially attractive since they are highly relevant for heart failure patients. Besides medical data, health economics information could provide insight into patients’ history of procedures and thus to help assessing cost-effectiveness of interventions. To align with the paradigm of patient empowerment and self-governance of medical data, enabling patients to voluntarily include their own data certainly holds potential. Large quantities of health-relevant data are collected with wearable sensors and consumer devices routinely now by many people including physical activity, number of steps, sleep quality and even physiological data like oxygen saturation or single-lead electrocardiograms that can be recorded by smart watches.

**Figure 6:**
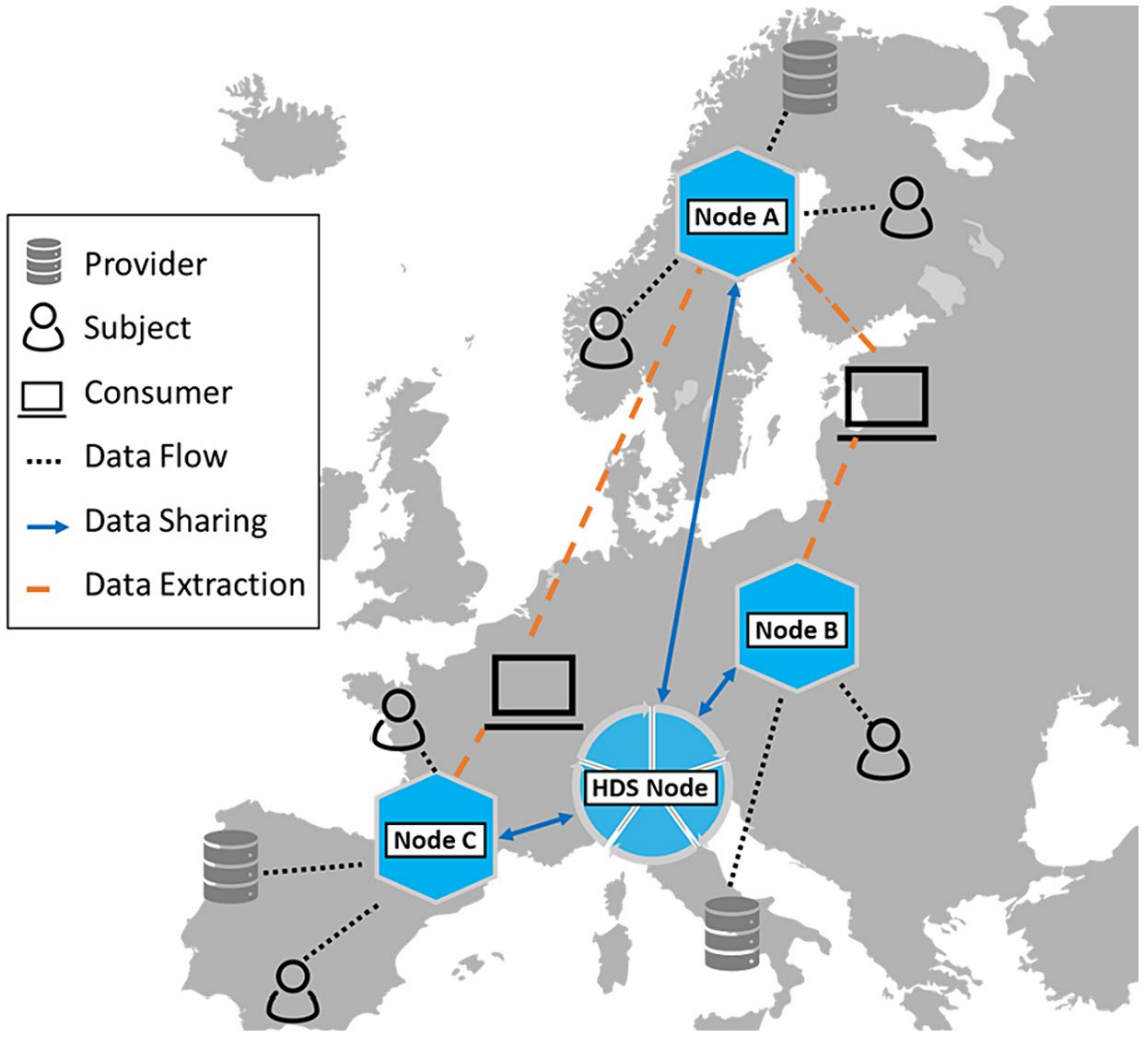
An HDS node can act as a national node in the context of a European data market and research infrastructure, such as currently being developed within the European Health Data Space.

Secondary use of health data might be regulated differently in individual countries or governance regions further complicating the issue of data sovereignty. Especially the transatlantic relationship has been strained by the overturning of both the *International Safe Harbor Privacy Principles* in 2015 (57) and the *EU-US Privacy Shield* in 2020 (58) agreements due to concerns of the Court of Justice of the European Union. However, the European Commission has recognized the potential of secondary use and aims to facilitate a common data space inside the European Union. The Commission has published several documents as part of its Data Strategy to work toward a European Health Data Space (EHDS). These concerted efforts are aiming for better utilization of data in both primary and secondary use and more convenience for patients in accessing health services abroad (59). The present work was inspired by this initiative and is intended to contribute to the evolution of the EHDS. Currently, data exchange and linkage policies can already be adapted to support various levels of record linkage across different jurisdictions. With this flexibility, HDS nodes could be linked to the EHDS and service interfaces to existing data space connector solutions such as the Eclipse Dataspace Connector (60) or the International Data Spaces Connector (61), as illustrated in Figure 6. Future work should also consider further development of the HDS node to adhere to specifications coming from initiatives like the EHDS and Gaia-X (62) and also keep different legislative frameworks in mind. Collaboration with similar frameworks like the *Personal Health Train* (24) could also prove fruitful for increasing data availability in the future. Furthermore, the capabilities of Blockchain technology to ensure data immutability could also be topic of future search as it would further increase trust in the system.

Architecture sustainability is always a concern in research projects like this because wide adoption of digital health solutions into regular healthcare settings is notoriously slow. Furthermore, the project-based funding and non-commercial setting of such systems make them inherently at risk of being not fully supported long-term. Many definitions for sustainability in the context of software exist (63). According to Venters et al. (64), sustainability describes a system’s *extensibility*, *interoperability*, *maintainability*, *portability*, *reusability*, *scalability*, and *usability*. We outlined that our infrastructure is portable and reusable by relying on common and platform-agnostic frameworks (e.g., Python). Further, we also described how we ensured interoperability by utilizing standard vocabulary (e.g., ICD-10, SNOMED, LOINC) and a suitable and commonly used common data model (OMOP CDM). With our PPRL methods, we also focused on the scalability and extensibility of the system by enabling vertical and horizontal linkage across different data sources. The greatest limitation toward scalability and extensibility remaining is the organizational coordination and data sovereignty concerns. To address this, future work could also focus on education and informing stakeholders about the benefits of such technology. Reference projects like the hereby described platform could aid this process. We addressed maintainability by aiming to minimize dependencies on third-party modules and relying on well-maintained open-source modules whenever possible. Since maintainability of our own core components is still a concern, we are also exploring options to potentially open-source parts of our code as well. This would open our developments to interested communities and improve maintainability by possibly increasing the amount of people interested in and working on the software. Usability is currently the least addressed aspect of sustainability in the HDS nodes. Although basic feedback from users (e.g., healthcare professionals, data scientists) has been implemented on occasion, systematic usability tests with stakeholders remain subject of future research. We recognize usability as a core requirement to aid the transition of stakeholders toward digital health solutions and have therefore included thorough usability testing in our development roadmap.

## 6 Conclusion

We have developed Health Data Space nodes to facilitate the secondary use of health data, which also support privacy-preserving record linkage across data sources to increase data availability. The HDS nodes provide sufficient flexibility to set up application specific infrastructures. With this concept, we realized and presented a pilot case study, including not only development but also deployment of a smart health ecosystem in a real-world infrastructure to establish the D4Health Heart Failure Registry for a routine care setting in Tyrol. With this infrastructure, data can be linked in a privacy-preserving way and be harmonized for interoperability. Preliminary functionality for collaborative feature engineering and model deployment have been tested in simple use cases. In conclusion, we consider these results as the foundation for future developments. Due to the modular architecture, the application of HDS nodes is not restricted to heart failure, but can be applied in various other scenarios.

We believe that such smart health ecosystems which support data management and MLOps and connect data from different health data spaces are the key to successful, efficient and sustainable secondary use of health data. Adhering to privacy standards is not only necessary from a with legal compliance perspective but also helps to improve overall acceptance and is, therefore, considered a must. With the presented case study, we hope to prove the feasibility of such systems and hope to inspire similar pioneering solutions for the upcoming work of building the European Health Data Space.

## Data availability statement

The data analyzed in this study is subject to the following licenses/restrictions: The clinical notes used for the NLP use case are considered medical data and therefore are not to be made public. Requests to access these datasets should be directed to martin.baumgartner@ait.ac.at.

## Ethics statement

The studies involving humans were approved by Ethics Commission of the Medical University of Innsbruck. The studies were conducted in accordance with the local legislation and institutional requirements. The participants provided their written informed consent to participate in this study.

## Author contributions

MB: Conceptualization, Data curation, Formal analysis, Investigation, Methodology, Software, Validation, Visualization, Writing – original draft, Writing – review & editing. KK: Conceptualization, Data curation, Investigation, Methodology, Project administration, Resources, Software, Supervision, Validation, Visualization, Writing – review & editing. AL: Conceptualization, Data curation, Investigation, Methodology, Software, Validation, Writing – review & editing. BJ: Conceptualization, Data curation, Investigation, Methodology, Software, Validation, Writing – review & editing. KD: Conceptualization, Investigation, Methodology, Resources, Supervision, Validation, Writing – review & editing. DH: Conceptualization, Funding acquisition, Investigation, Methodology, Project administration, Resources, Supervision, Writing – review & editing. FW: Investigation, Methodology, Software, Writing – review & editing. LD: Investigation, Methodology, Software, Writing – review & editing. RM-O: Conceptualization, Funding acquisition, Methodology, Project administration, Resources, Supervision, Validation, Writing – review & editing. SN: Conceptualization, Funding acquisition, Project administration, Resources, Validation, Writing – review & editing. GSl: Conceptualization, Funding acquisition, Methodology, Project administration, Resources, Supervision, Validation, Writing – review & editing. SP: Conceptualization, Funding acquisition, Methodology, Project administration, Resources, Supervision, Validation, Writing – review & editing. LB: Conceptualization, Investigation, Methodology, Supervision, Validation, Writing – review & editing. BP: Conceptualization, Funding acquisition, Investigation, Methodology, Project administration, Resources, Supervision, Validation, Writing – review & editing. GP: Conceptualization, Formal analysis, Funding acquisition, Investigation, Methodology, Project administration, Resources, Supervision, Validation, Writing – review & editing. GSc: Conceptualization, Data curation, Formal analysis, Funding acquisition, Investigation, Methodology, Project administration, Resources, Software, Supervision, Validation, Visualization, Writing – review & editing.
